# A Regional NWP Tropospheric Delay Inversion Method Based on a General Regression Neural Network Model

**DOI:** 10.3390/s20113167

**Published:** 2020-06-03

**Authors:** Lei Li, Ying Xu, Lizi Yan, Shengli Wang, Guolin Liu, Fan Liu

**Affiliations:** 1 College of Geodesy and Geomatics, Shandong University of Science and Technology, Qingdao 266590, China; lei6lei22@126.com (L.L.); ylz_email@163.com (L.Y.); 18700846917@163.com (G.L.); 18292005018@163.com (F.L.); 2 Key Laboratory of Geomatics and Digital Technology of Shandong Province, Shandong University of Science and Technology, Qingdao 266590, China; 3 Institute of Ocean Engineering, Shandong University of Science and Technology, Qingdao 266590, China; shlwang@sdust.edu.cn

**Keywords:** GNSS, NWP, tropospheric delay, GRNN

## Abstract

Tropospheric delay is a major error source that affects the initialization and re-initialization speed of the Global Navigation Satellite System’s (GNSS) medium-/long-range baseline in Network Real-Time Kinematic (NRTK) positioning. Fusing the meteorological data from the Numerical Weather Prediction (NWP) model to estimate the zenith tropospheric delay (ZTD) is one of the current research hotspots. However, research has shown that the ZTD derived from NWP models is still not accurate enough for high-precision GNSS positioning applications without the estimation of the residual tropospheric delay. To date, General Regression Neural Network (GRNN) has been applied in many fields. It has a high learning speed and simple structure, and can approximate any function with arbitrary precision. In this study, we developed a regional NWP tropospheric delay inversion method based on a GRNN model to improve the accuracy of the tropospheric delay derived from the NWP model. The accuracy of the tropospheric delays derived from reanalysis data of the European Center for Medium-Range Weather Forecasts (ECMWF) and the US National Centers for Environmental Prediction (NCEP) was assessed through comparisons with the results of the International GPS Service (IGS). The variation characteristics of the residual of the ZTD inverted by NWP data were analyzed considering the factors of temperature, humidity, latitude, and season. To evaluate the performance of this new method, the National Center Atmospheric Research (NCAR) troposphere data of 650 stations in Japan in 2005 were collected as a reference to compare the accuracy of the ZTD before and after using the new method. The experimental results showed that the GRNN model has obvious advantages in fitting the NWP ZTD residual. The mean residual and the root mean square deviation (RMSD) of the ZTD inverted using the method of this study were 9.5 mm and 12.7 mm, respectively, showing reductions of 20.8% and 19.1%, respectively, as compared to the standard NWP model. For long-range baseline (155 km and 207 km), the corrected NWP-constrained RTK showed a reduction of over 43% in the initialization time compared with the standard RTK, and showed a reduction of over 24% in the initialization time compared with the standard NWP-constrained RTK.

## 1. Introduction

Network Real-Time Kinematic (NRTK) positioning with instantaneous ambiguity resolution (AR) is currently a popular technique for real-time precise positioning using carrier phase observations. The AR of the baselines between the reference station and user station is a key technical factor in Real-Time Kinematic (RTK) positioning [1]. The convergence of the AR or the initialization/re-initialization time in NRTK is significantly affected by the atmospheric delay over medium-/long-range baselines. As Global Navigation Satellite Systems (GNSS) provide dual or even multiple frequency signals, ionospheric delay can be removed from the combinations of different frequencies. As was reported in our previous paper [2], we developed a new ionosphere-free AR method for long-range baselines that can eliminate the ionospheric delay in the ambiguity search stage. In this study, we focus on the tropospheric delay.

Tropospheric delay can be divided into two parts: dry delay and wet delay. Generally, the former can be inverted accurately by the appropriate models. The latter accounts for only 10% of the total delay, but it is difficult to model using surface meteorological data. In general, there are four methods that can be used to reduce the effect of the tropospheric delay in GNSS precise data processing. The first method involves using empirical models to estimate the zenith tropospheric delay (ZTD) [3,4,5]. It is indicated that the accuracy of ZTD inversion by the experience model is about 5 cm and the maximum error is up to 20 cm. The second method is double differencing. In the relative positioning of the shorter baselines, most tropospheric errors can be eliminated by double differencing, and the residuals are ignored in the data processing. However, the effect of the spatially correlated residuals cannot be cancelled out for longer baselines, so they are treated as an indispensable part of the positioning process [5]. The third method, which is also the most accurate method, involves estimating tropospheric delays using GNSS measurements directly, together with other unknown parameters, e.g., station coordinates and ambiguities [6,7,8]. However, the convergence time for all existing algorithms is relatively long (typically 30–60 min) [9]. The last method involves using NWP reanalysis or prediction data to retrieve the ZTD. The products of the NWP are the three-dimensional meteorological parameters for a given area and point in time. These meteorological parameters from the NWP can thus be used to derive the tropospheric delay at any location and at any time within the area of the NWP model.

In recent years, many research institutes have provided numerical forecast data for a variety of meteorological observations. At present, the European Center for Medium-Range Weather Forecasts (ECMWF) and the United States National Centers for Environmental Prediction (NCEP) data are commonly used. Meanwhile, with the development of Earth observation technology, the accuracy and space-time resolution of these data has been continuously improved. Moreover, several tropospheric models have been developed using ECMWF or NCEP datasets, such as the UNB3 model [10], Trop Grid model [11], GPT2 model [12], EGNOS model [13], and SSIEGNOS model [14]. In recent years, several studies have been conducted to retrieve the ZTD derived from NWP reanalysis or forecast datasets, and to prove their advantages. Andrei et al. analyzed the International GPS Service (IGS) ZTD solutions of 18 globally distributed IGS stations over a 1.5 year period, and their results indicated that the ZTD derived from the NWP model-global data acquisition system (GDAS) agreed with the IGS ZTD solutions at a 3.0 cm root mean square (RMS) error level with biases of up to 4.5 cm [15]. Chen et al. calculated the ZTD by the integration of ECMWF/NCEP pressure-level data of 28 GPS stations in China for 1 year. The results showed that the error of the integration method was 1–3 cm less than that of the Saastamoinen model method [16]. Jensen et al. used NWP zenith delays in a static positioning process to correct for the tropospheric delay, and the 3D position accuracy was improved for 15 out of the 26 baselines processed when the NWP approach was used instead of the global Saastamoinen model [5]. GhoddousiFard et al. proved the good accuracy of the zenith hydrostatic delay (ZHD) derived from the NWP models, but the accuracy of the zenith wet delay (ZWD) derived from these models was usually far less than that of the ZHD, especially in low-altitude regions [17]. Yong et al. analyzed GPS RTK performance using external National Oceanic and Atmospheric Administration (NOAA) real-time tropospheric corrections integrated with a multiple reference station approach in three geographical regions in the U.S. National Geodetic Survey network of Continuously Operating Reference Stations (CORS), and showed a 3% improvement compared with the modified Hopfield model [18]. Lu et al. used tropospheric parameters from the ECMWF for Precise Point Positioning (PPP), and they found that both the convergence time and positioning accuracy were improved obviously for the north, east, and vertical components [19]. Yuan et al. assessed the Zenith Hydrostatic Delay (ZHD), the Zenith Wet Delay (ZWD) and mapping functions derived from the ECMWF data. Test results showed that the Zenith Total Delays (ZTD) derived with the NWP data were shown to agree well with the tropospheric delay product from the Center for Orbit Determination Europe (CODE). Root mean square (RMS) errors associated with these ZTD estimates were < 10 mm at all 28 stations [20]. Jiang et al. evaluated the performance of ZTD derived from ECMWF reanalysis data over China using 219 GNSS stations of the Crustal Movement Observation Network of China (CMONOC) covering the period from 2015 to 2016. Test results showed that the average bias and Root Mean Square (RMS) of ZTD in model method are 7.97 mm, 39.25 mm [21]. Nevertheless, the ZTD derived from NWP models is still not accurate enough for high-precision GNSS positioning applications without the estimation of the tropospheric delay residual. 

In 1988, Moody and Darken introduced radial basis functions (RBF) into neural network operations [22], and the RBF neural network model began to enter a boom period. Scholars have conducted extensive research on RBF neural network models. The basic idea of the RBF network is outlined as follows: for input and output samples, the input samples are transformed from a low-dimensional space to a high-dimensional space by nonlinear transformation, and then the output is obtained by linear transformation from the hidden layer to the output layer (weighted average sum). In 1991, Specht proposed General Regression Neural Network (GRNN), a variant of the RBF neural network [23]. GRNN is based on nonlinear regression theory for function estimation, and calculates the network output vectors according to the principle of maximum probability. Compared with RBF neural network, the training process of GRNN is more convenient, especially suitable for solving curve fitting problems, and has been widely used in various disciplines and engineering fields. 

In this study, we focus on developing a regional NWP tropospheric delay inversion method based on a GRNN model to improve the accuracy of the tropospheric delay. The quality of tropospheric delay parameters retrieved from the ECMWF and NCEP analysis is assessed by comparison with the IGS final tropospheric delay products. The variation characteristics of the residual of the ZTD inverted by NWP data are analyzed, considering the factors of temperature, humidity, latitude, and season. The performance of the GRNN model in NWP tropospheric delay estimation is evaluated based on the National Center Atmospheric Research (NCAR) troposphere data and ECMWF reanalysis data of 650 stations in Japan in 2005.

This article is organized as follows: experimental data and the method used to calculate the ZTD with NWP data are introduced in Section 2. The accuracy of the ZTD inverted by ECMWF and NCEP reanalysis data is also compared in this section. The variation characteristics of the residual of the ZTD is analyzed in Section 3, and a regional NWP tropospheric delay inversion method based on a GRNN model is proposed. To evaluate the performance of this model, experiments and analysis are conducted and are described in Section 4. The conclusions are given in Section 5. 

## 2. Accuracy Comparison of ZTD Inverted by ECMWF/NCEP Reanalysis Data

### 2.1. Experimental Data

ECMWF reanalysis data from 2013 to 2016 and NCEP reanalysis data of 2016, the high-precision troposphere products of 100 globally distributed International GPS Service (IGS) stations from 2013 to 2016, and the NCAR troposphere data for 650 stations in Japan in 2005 were collected for this study.

ECMWF is an organization that aims to provide users with global numerical weather forecasts, and has provided decades of ECMWF reanalysis data. These reanalysis data are important resources for atmospheric and oceanographic studies. In this paper, the pressure-level data of the ERA-Interim (January 1979–present) product from ECMWF were used. The horizontal resolution of the data is 0.75° × 0.75° (the data of 0.125° × 0.125° used in this study were downloaded directly from the official website and refer to the interpolated grid). The vertical resolution is 37 pressure levels, the top pressure is 1 hpa, and the time resolution is 6 h (0, 6, 12, 18 UTC) [24]. The stratified data used in this study included pressure, geopotential, temperature, and relative humidity. The data URL can be referred to [24]. The NCEP reanalysis project began in 1991, and was the product of the NCEP FNL (Final) operational model global tropospheric analyses (continuing from July 1999) were used. The horizontal resolution of the data is 1° × 1°, the vertical resolution is 26 mandatory (and other pressure) levels, the top pressure is 10 hpa, and the time resolution is 6 h (0, 6, 12, 18 UTC) [25]. the NCEP climate data assimilation system project. In this paper, the layered meteorological data of the stratified data used in this study included pressure, geopotential height, temperature, and relative humidity. The data URL can be referred to [25]. It is worth mentioning that the resolutions of the ECMWF and NCEP reanalysis data selected in this paper are the highest resolutions that can be downloaded from the corresponding weather forecast center websites, so the resolutions of the two kinds of reanalysis data are different.

Since 2007, The IGS center has regularly provided ZTD products with a precision of 1.5–5.0 mm and a time resolution of 5 min for some IGS stations in the world [26]. As compared with the previous method—specifically, that the IGS started the production of ZTD estimates by combining the contributions of ZTD estimates from several IGS Analysis Centers (Acs) in 1997—this product accelerates the update cycle and can be better used in meteorology research [26]. Meanwhile, the NCAR troposphere data is derived from GPS data with a time resolution of 2 h. It can provide high-precision tropospheric data for more sites than the IGS ZTD, and is widely used in various climate studies [27].

### 2.2. ZTD Inversion Method with NWP Data

The integral method and the Saastamoinen method [16] are usually used to calculate the ZTD by NWP data, though the integral method is more accurate [16]. Therefore, the integral method is used in this paper. Equation (1) establishes the relationship between refractivity and meteorological parameters; the refractivity can be calculated as follows [15,28]:

(1) $$N=k_{1}\frac{p}{T}+(k_{2}\frac{e}{T}+k_{3}\frac{e}{T^{2}})=N_{dry}+N_{wet}$$

where $K_{1}=77.604\;K/\mathrm{mbar}$, $K_{2}=64.79\;K/\mathrm{mbar}$, $K_{3}=377600.0\;K^{2}/\mathrm{mbar}$ and $k_{1},\;\;k_{2},\;\;k_{3}$ represent the air refractivity parameters [28]. *T* is the temperature (unit: K), *p* is the atmospheric pressure (unit: mbar), *e* represents the water vapor pressure (unit: mbar), $N_{dry}$ represents the refractivity of dry gas, and $N_{wet}$ represents the refractivity of wet gas.

After calculating the total refractivity, the ZTD (unit: m) is derived by integrating the refractivity:

(2) $$ZTD=10^{-6}\int_{H}NdH=10^{-6}\sum_{i=1}^{n}N_{i}\Delta H_{i}.$$

where *N* is the average value of refractivity calculated by the upper and lower meteorological parameters in the integral region, H is the height from the layer closest to the target station to the top level of the meteorological data (unit: m), n is the level of integration, $N_{i}$ is the atmospheric refractivity in the *i*th integral area, and $\Delta H_{i}$ is the height of the *i*th integral area.

Since the IGS stations and the ECMWF/NCEP grid points are usually not collocated and have different altitudes, interpolation is necessary [29]. To realize the interpolation, four ECMWF/NCEP grid points surrounding the target point are selected. The altitude difference corrections are gained by Gaussian function, and then the ZTD of the target point is calculated by bilinear interpolation [29]. The Gaussian function used for altitude difference correction is obtained by fitting the ZTD and the altitude curve. The fitting formula of the Gaussian function is as follows:

(3) $$ZTD_{H}=a\cdot \exp\left\{-\frac{{\left(H-b\right)}^{2}}{c^{2}}\right\}$$

where ${\mathrm{ZTD}}_{H}$ (unit: m) is the *ZTD* value at the target height *H; a, b*, and *c* are the Gaussian function coefficients. Moreover, the atmosphere above the highest layer of the NWM gives a non-negligible contribution to the *ZTD*. To solve this problem, we input the meteorological data of the top layer into the Saastamoinen model [4] to calculate the zenith delay, and the result is added to the integral delay to obtain the final *ZTD*.

### 2.3. Accuracy Comparison of ZTD Inverted from ECMWF/NCEP Reanalysis Data

**Figure 1:**
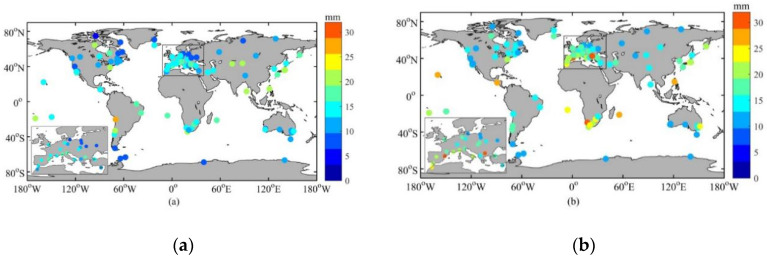
Distribution of 100 IGS stations, the mean residual (**a**) and RMSD (**b**) of ZTD inverted by ECMWF) reanalysis data (2016). The color of the dots in (**a**) and (**b**) represent the numerical values of the ZTD mean residual and RMSD of the ZTD for each station, respectively.

The ZTDs for 100 globally distributed IGS stations were inverted by using the ECMWF and NCEP reanalysis data of 2016, separately. The distribution of IGS stations is shown in Figure 1. Taking the high-accuracy troposphere products of IGS stations as the reference, the mean value, minimum value, and maximum value of the residual and the RMSD of the ZTD inverted by two kinds of reanalysis data are shown in Table 1. The mean value, minimum value, and maximum value of the ZTD residual were calculated in this study based on the absolute value of the ZTD residual obtained daily at 18 UTC over 1 year. We employed the absolute value in order to observe the residual clearly. If the absolute value is not taken, the average value will approach 0, and the minimum and maximum values are not easy to compare. The RMSD calculation formula is as follows:

(4) $$RMSD=\sqrt{\frac{1}{M}\overset{M}{\underset{t=1}{\sum }}{\left(observed_{t}-predicted_{t}\right)}^{2}}$$

where $observed_{t}$ is the ZTD residual and $predicted_{t}$ is the true ZTD residual value (0), *t* is the day of year, *M* is the total number of the days.

As can be seen from Table 1, the mean residuals inverted by ECMWF and NCEP reanalysis data are about 13–17 mm; the mean residual inverted by ECMWF data is slightly smaller than that inverted by NCEP data. Both the mean RMSDs inverted by the two data are more than 17 mm, and the mean RMSD inverted by ECMWF data is obviously smaller than that inverted by NCEP data. Specifically, the maximum mean residual of the ZTD inverted by NCEP reanalysis data reaches 29.7 mm and the maximum RMSD is 33.9 mm. This reflects that (1) the accuracy of the ZTD inverted by using the ECMWF reanalysis data is higher than that inverted using NCEP data, and (2) the accuracy of the ZTD inverted by the NWP is not high enough to be used in precise positioning directly. In addition, the precision of the NWP forecast data is lower than that of the reanalysis data, and it also cannot be directly used for high-precision positioning.

## 3. Regional NWP Tropospheric Delay Inversion Method Based on GRNN Model 

### 3.1. Analysis of Influencing Factors of ZTD Residuals Inverted by NWP Model

According to the above analysis, the accuracy of the ZTD inverted by using the ECMWF data is higher than that inverted by NCEP data. As a consequence, the ECMWF data were chosen as the experimental data in this paper. Since the mean residual and RMSD of the ZTD by using the NWP model can reach more than 30 mm, it will affect the initialization of PPP and RTK. To overcome this issue, firstly, the change law of the residual of the ZTD inverted using ECMWF data was analyzed with temperature, humidity, latitude, season, and other factors. Figure 1 shows the distribution of the 100 IGS stations, the mean residual (a) and RMSD (b) of the ZTD inverted by the 2016 ECMWF reanalysis data, with some areas enlarged. The characteristics of mean residual and RMSD can be seen roughly from this figure. In general, the mean residual and RMSD of the ZTD in higher latitudes are smaller than those in lower latitudes, and the mean residual and RMSD of the ZTD in inland areas are smaller than those in offshore areas. Table 2 shows the mean value, minimum value, and maximum value of the residual and RMSD of the ZTD inverted by ECMWF reanalysis data at different latitudes. The ranges of low-latitude, mid-latitude, and high-latitude regions are 0°~30°, 30°~60°, and 60°~90° (absolute values), respectively. As can be seen from this table, the mean residual of the low-latitude, mid-latitude, and high-latitude regions are 17.3 mm, 13.6 mm, and 10.2 mm, respectively. The RMSD of the low-latitude, mid-latitude, and high-latitude regions are 21.7 mm, 16.9 mm, and 11.8 mm, respectively. As a result, the accuracy of the ZTD inverted by ECMWF data increases as the latitude increases. In Figure 1, especially in the enlarged region, it can be seen that the accuracy of the ZTD in inland areas is higher than that in offshore areas. The possible reasons for this would be: (1) the temperature in low-latitude regions is usually higher than that in high-latitude regions. The higher the temperature, the more violent the atmosphere and the harder it is to calculate the ZTD using meteorological data [30,31]. Therefore, high temperature may reduce the accuracy of the ZTD inverted by NWP. (2) A higher amount of water vapor can be observed in the area near water, and water vapor has an impact on the accuracy of ZTD inversion [30].

The tropospheric delay can be expressed as a function of the satellite elevation angle and altitude of the GPS receiver, depending on the atmospheric pressure, temperature, and water vapor pressure [32]. The hot and wet conditions are closely related to the tropospheric effect, especially the wet components, which are approximately proportional to the content of water vapor in the troposphere [17]. Moreover, a strong seasonal and interannual variability of mesoscale fluctuations of the zenith tropospheric delay of decimeter radio waves was detected in [31]. For further study, the influence of temperature and humidity on the accuracy of the ZTD and the variation curve of the time series of the ZTD residual are discussed.

**Figure 2:**
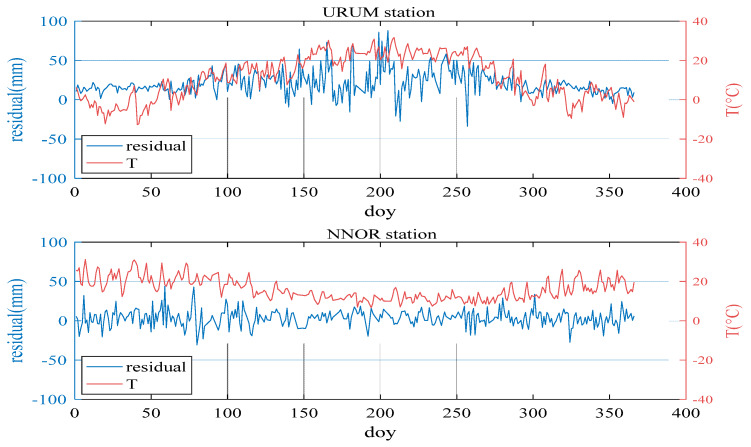
ZTD residual (blue line) and temperature (red line) of URUM station and NNOR station.

**Figure 3:**
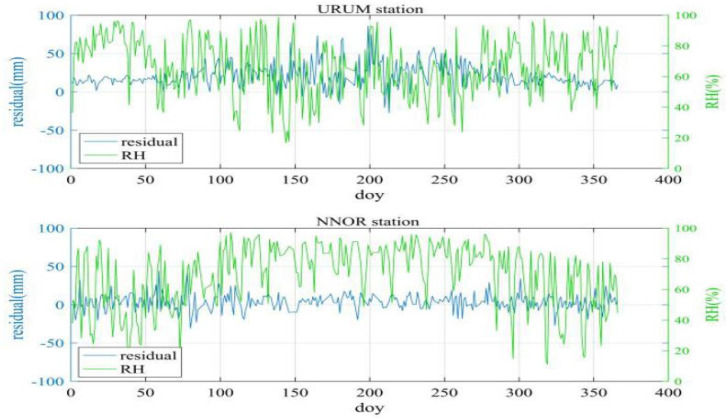
ZTD residual (blue line) and relative humidity (green line) of URUM station and NNOR station.

The temperature and relative humidity time series of two stations in 2016 (1 year) were plotted. The two stations are URUM station (43.59° N, 87.63° E) and NNOR station (31.05° S, 116.19° E). The results are indicated in Figure 2 and Figure 3. It can be seen from these figures that the temperature and relative humidity have a high correlation with the residual of the NWP ZTD.

As shown in Figure 2, when the temperature is high, the residual of the ZTD is relatively large. URUM station is in the northern hemisphere. Thus, when the doy is between 150 and 250, in summer, the temperature is mostly above 20 °C and the ZTD residual is relatively large and fluctuates violently. Moreover, when the temperature is below 10 °C, in winter, even though the temperature is dramatically changed, the ZTD residual is small. Unlike URUM station, NNOR station is in the southern hemisphere. Thus, when the doy is between 150 and 250, the temperature is about 10 °C, in winter, and the ZTD residual is relatively small. The ZTD residual is also large in the summer season.

As shown in Figure 3, in summer, the relative humidity fluctuates violently, and the residual of the ZTD also fluctuates accordingly. In winter, the relative humidity fluctuates little, and the ZTD residual also fluctuates little. In short, the curve of relative humidity and the ZTD residual are similar.

**Figure 4:**
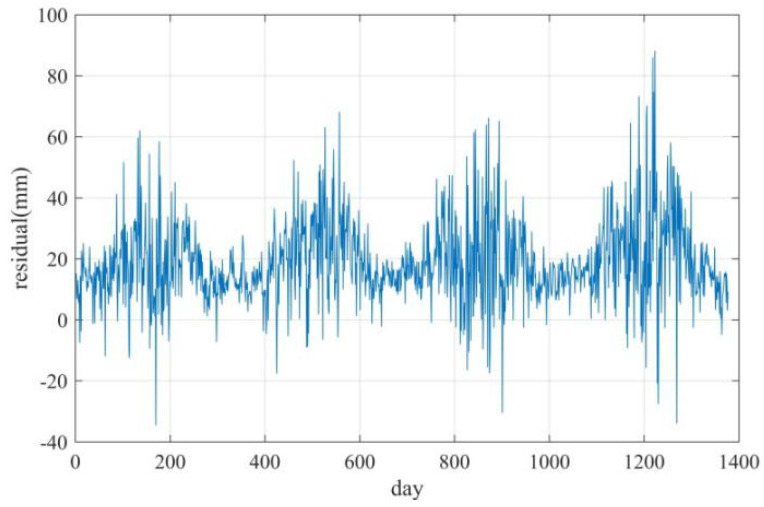
ZTD residual for URUM station (2013–2016).

To discuss the seasonal variation of the ZTD residual, four consecutive years’ (2013–2016) ZTD of the IGS URUM station were inverted using ECMWF reanalysis data. The ZTD residual curve is shown in Figure 4. It can be seen that the variation of the ZTD residual has characteristics of interannual variation, and the annual variation of the residual curve is similar.

### 3.2. Regional NWP Tropospheric Delay Inversion Method Based on GRNN Model

As can be seen in [32], it is clear that the a priori troposphere model cannot effectively eliminate the residual tropospheric delay. To reduce the residual of tropospheric delay inverted by using NWP data, appropriate model is necessary. As analyzed in Section 3.1, the factors of temperature and relative humidity affect the ZTD residual significantly. The overall distribution of the atmosphere is uneven and irregular, and it is difficult to establish a global tropospheric delay residual correction model. Based on these characteristics, a regional NWP tropospheric delay inversion method based on a GRNN model is proposed in this study.

GRNN was first proposed by Specht [23]. It belongs to the radial basis function networks. The pattern layer of GRNN generally adopts a Gaussian function, which can approximate a continuous value with arbitrary precision. Each neural unit in the pattern layer has a basis function, which linearly combines these basis functions through the weights. Infinite approximation makes the output of the neural network approach a certain value or causes it to no longer change, so that the GRNN model is stabilized. The GRNN model draws the function estimate directly from training data, and does not need an iterative training procedure; thus, GRNN does not have a local minimum issue.

**Figure 5:**
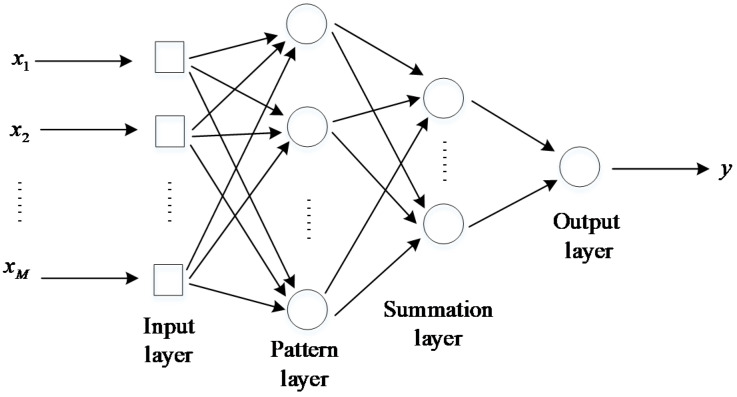
The structure of General Regression Neural Network.

The GRNN structure is presented in Figure 5. The training set includes values of inputs *x*, each with a corresponding value of an output *y*. In general, GRNN consists of four layers: an input layer, a pattern layer, a summation layer, and an output layer. The input layer provides all of the measurement variables *x* to all of the neurons on the pattern layer. The pattern layer is dedicated to one exemplar or one cluster center. The neurons of the summation layer are divided into two categories. One calculates the algebraic sum of the neurons in the pattern layer, which is called the denominator unit, and the other calculates the weighted sum of the neurons in the pattern layer, which is called the molecular unit. The output layer merely divides the denominator unit by the molecular unit to yield the desired estimate of *y*. Further details about GRNN can be found in [33,34]. In this study, MATLAB software (R2016a) was used to implement GRNN.

**Figure 6:**
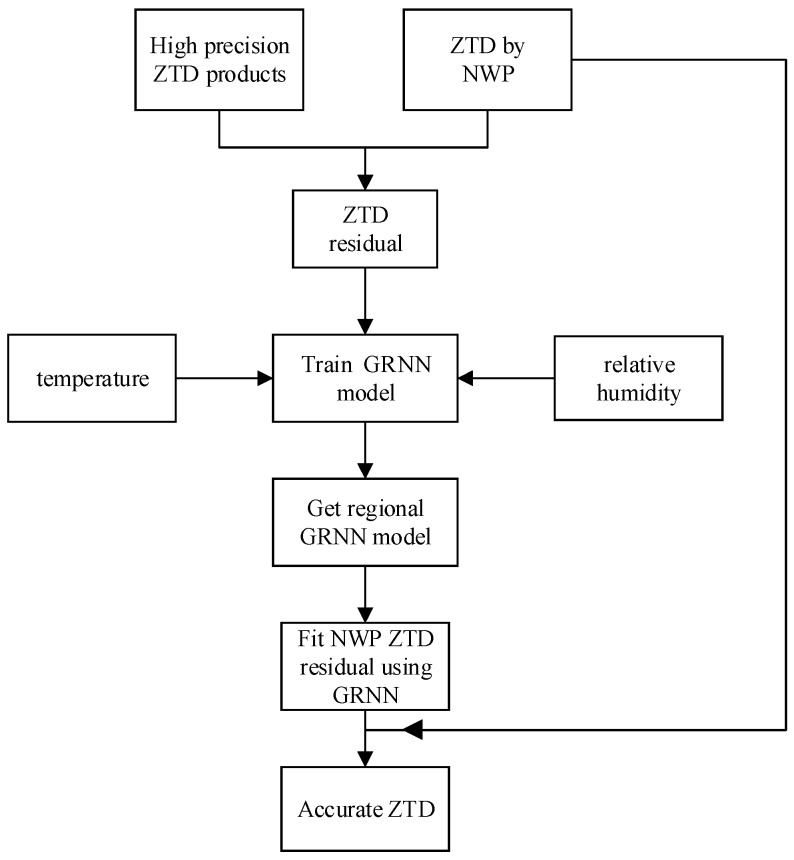
The flow chart of inverting the ZTD by the regional NWP tropospheric delay inversion method based on a GRNN model.

In this method, stations that serve as training data should be selected firstly, and these stations should satisfy the following two points: (1) the precise tropospheric delay of the station must be known (2) stations should be distributed as evenly as possible to prevent over-fitting or under-fitting of the GRNN model. Secondly, the GRNN model is established by using the selected training station data. The parameters needed for the model include the temperature, relative humidity and NWP ZTD residuals of the training station. The GRNN model of the training station area is obtained after parameter training. The NWP ZTD residual of uncertain stations can be obtained by using the GRNN model. In this process, the inputs x are the temperature and relative humidity of the stations, and the output y are the NWP ZTD residuals. Finally, the accurate ZTD of uncertain stations can be obtained by subtracting the fitting residual. Taking seasonal factors of the NWP ZTD residuals into account, this method selects 1 year of data for each experimental station for testing. Figure 6 is the flow chart of inverting the ZTD by the regional NWP tropospheric delay inversion method based on a GRNN model.

## 4. Experiments and Analysis

### 4.1. The Regional NWP Tropospheric Delay Inversion Method Based on a GRNN Model Analysis

**Figure 7:**
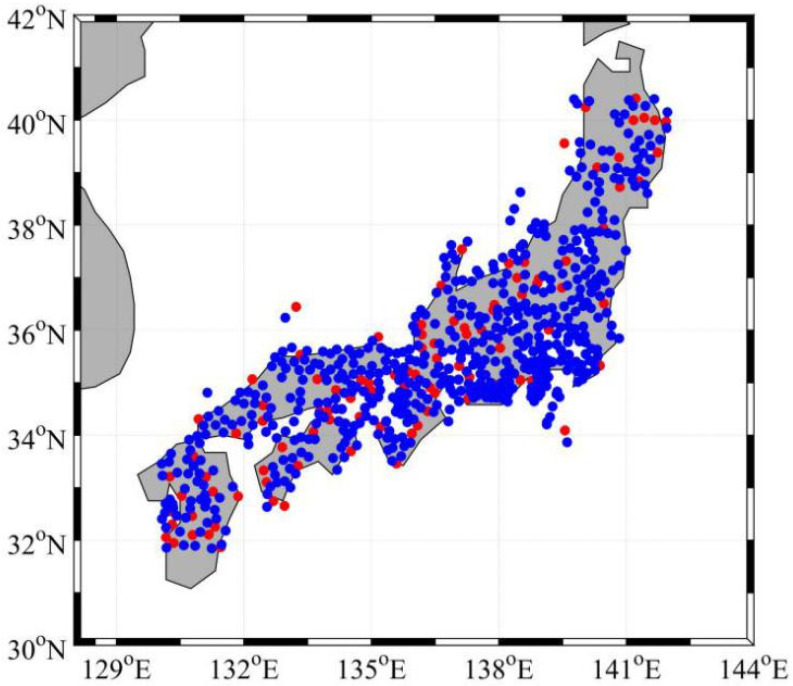
Distribution of the 650 stations in Japan. The red dots represent the 100 stations providing training data, and the blue dots represent the 550 stations providing testing data.

In this section, the validity and accuracy of the regional NWP tropospheric delay inversion method based on a GRNN model is evaluated. The NCAR troposphere data and ECMWF reanalysis data of 650 stations in Japan in 2005 were collected, 100 stations that are evenly distributed were selected as training samples, and the remaining 550 stations were tested. The distribution of the 650 stations in Japan is presented in Figure 7. The range of the experimental area is 32° N~40° N, 130° E~142° E.

**Figure 8:**
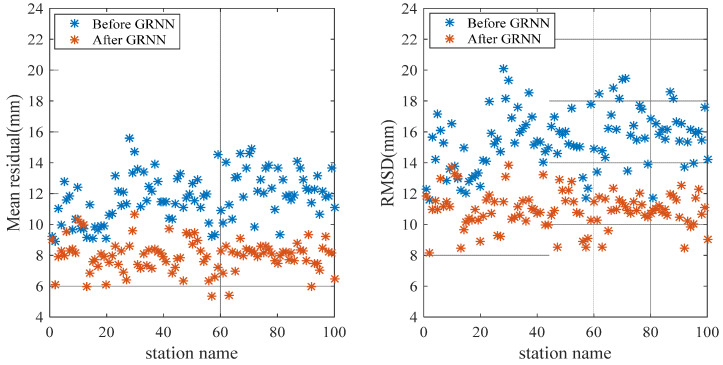
Comparison of the mean residual and RMSD before and after using the GRNN model for 100 training stations in 2005.

**Figure 9:**
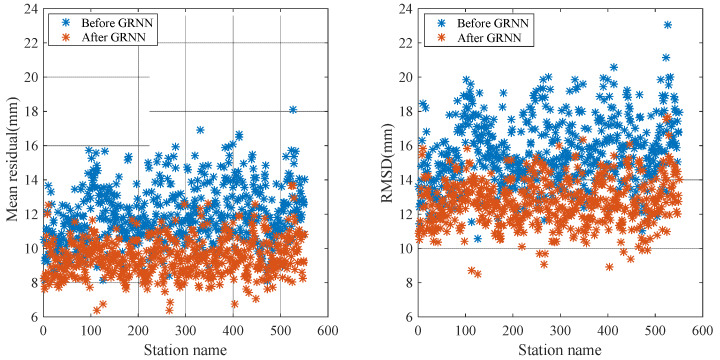
Comparison of mean residual and RMSD before and after using the GRNN model for 550 test stations in 2005.

Figure 8 and Figure 9 show the comparison of the mean residual and RMSD before and after using the GRNN model for 100 training stations and 550 test stations, respectively. Before weakening the ZTD residual by using the GRNN model, the mean ZTD residual and RMSD mainly fluctuated at 12 and 16 mm, respectively. Both the mean ZTD residual and RMSD decreased obviously after weakening the ZTD residual using the GRNN model. 

**Figure 10:**
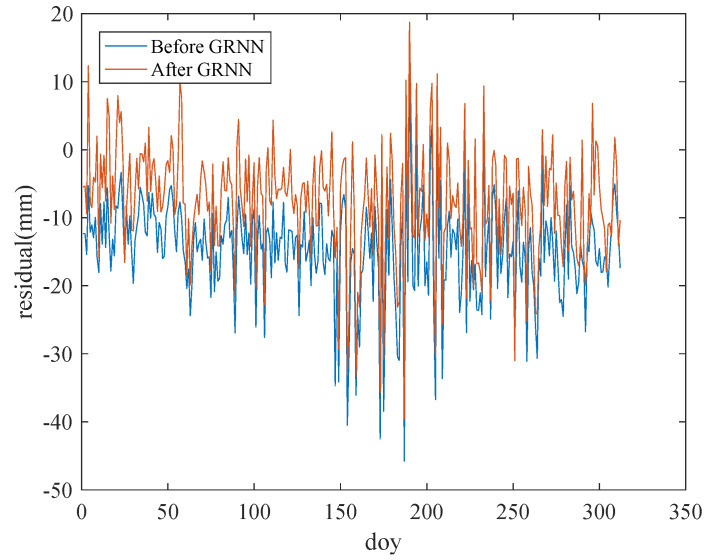
Comparison of residual before and after using the GRNN model for one of the test stations.

**Figure 11:**
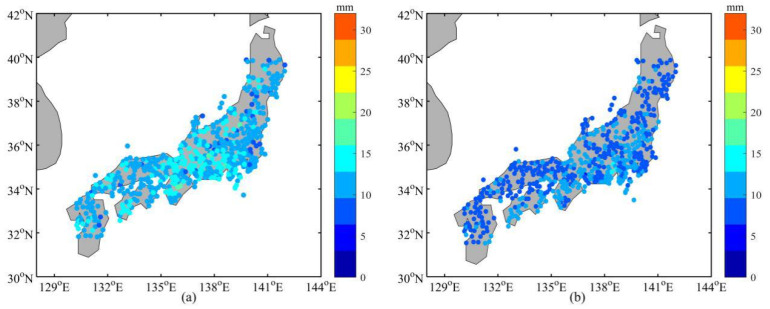
Comparison of the ZTD mean residual before (**a**) and after (**b**) using the GRNN model for 550 test stations in 2005. The color of the dots represents the numerical value of mean residual for each station.

Figure 10 is the comparison of the residual before and after using the GRNN model for one of the test stations. As shown in Figure 10, the residual decreased after using the GRNN model. To clearly show the variation of the NWP ZTD residuals before and after using the GRNN model for each station, Figure 11 illustrates the ZTD mean residual before (a) and after (b) using the GRNN model for the 550 test stations in 2005. The mean residual reduction at each station can be clearly seen in Figure 11. Table 3 lists the residual statistics before and after using the GRNN model for selected stations; Table 4 shows the RMSD and standard deviation (SD) of the residual before and after using the GRNN model for selected stations. As can be seen from Table 3, the mean residuals of 100 training stations and 550 test stations before using the GRNN are 11.8 mm and 12.0 mm, respectively. The mean residuals after using the GRNN are 8.0 mm and 9.5 mm, respectively, showing reductions of 32.2% and 20.8%, respectively. As can be seen from Table 4, the RMSDs of 100 training stations and 550 test stations before using the GRNN are 15.5 mm and 15.7 mm, respectively. The RMSDs after using the GRNN are 10.9 mm and 12.7 mm, respectively, showing reductions of 29.7% and 19.1%, respectively. The SD of test stations after using the GRNN exhibited no obvious change, reflecting that the GRNN model only has a slight impact on ZTD variation. Since both the residual and RMSD decreased, the accuracy of the ZTD with the application of the GRNN model is improved.

### 4.2. GPS RTK Results

#### 4.2.1. GPS Medium-/Long-Range RTK Algorithm Constrained with NWP Model

For medium-/long-range baselines, measurements errors (including ionospheric delay, tropospheric delay, phase wind-up, relativistic effects, tides) should be considered due to the large effect of the errors on the double difference ambiguity resolution and positioning. The tides and other geophysical related errors can be estimated using methods and models [35]. As a result, the tropospheric delay and ionospheric delay are the main errors that affect the performance of GPS medium/long-range RTK. In this study, the ionospheric delay was estimated by using the method we developed in [2]. After the ionospheric delay and other measurement errors are removed, the GPS RTK processing model can be expressed as follows:

(5) $$\left[\begin{array}{c} V_{\Delta P}\\ V_{\Delta \phi } \end{array}\right]=\left[\begin{array}{c} \begin{array}{ccc} A & C & \;0 \end{array}\\ \begin{array}{ccc} A & C & \lambda_{i}\cdot E \end{array} \end{array}\right]\left[\begin{array}{c} B\\ T\\ \Delta N_{i} \end{array}\right]-\left[\begin{array}{c} \Delta P_{i}\\ \Delta \phi_{i} \end{array}\right]$$

where $V_{\Delta P}$ and $V_{\Delta \phi }$ are the residual vectors of the double difference pseudo-range and phase observation; *i* represents the frequency; *A* is the design matrix; *C* is the coefficient corresponding to *T*, and *C* is the unit matrix here; $\lambda_{i}$ is the wavelength of the corresponding GNSS observation; *E* is the unit matrix; *B* is the vectors of unknown parameters of baseline components. *T* is double difference slant total delay, $\Delta N_{i}$ is the GNSS double difference ambiguity, $\Delta P_{i}$ and $\Delta \phi_{i}$ are the double difference GNSS pseudo-range and phase observation, respectively.

The double difference slant total delay *T* can be described as the sum of the double difference hydrostatic and non-hydrostatic/wet components [5]:

(6) $$T=\Delta_{ZHD}\cdot {\mathrm{Mf}}_{h}+\Delta_{ZWD}\cdot {\mathrm{Mf}}_{\mathrm{nh}}$$

where $\Delta_{ZHD}$ and $\Delta_{ZWD}$ are the double difference zenith hydrostatic and non-hydrostatic/wet delays, respectively. ${\mathrm{Mf}}_{h}$ and ${\mathrm{Mf}}_{\mathrm{nh}}$ are the corresponding hydrostatic and non-hydrostatic mapping function, and we used the GMF Global Mapping Function (GMF) in this research [36].

To investigate the performance of the new model in GNSS positioning, three RTK scenarios including the standard RTK, the NWP-constrained RTK and the corrected NWP-constrained RTK are carried out in this study. For tropospheric delay, in the standard RTK processing, the hydrostatic delay $\Delta_{ZHD}$ is calculated by using the Hopfield model in this study. Owing to the high variability of the water vapor distribution, the wet delay $\Delta_{ZWD}$ is estimated as an unknown parameter in the adjustment together with the other parameters, such as the station coordinates and the ambiguities. As with the standard RTK, we remove the hydrostatic delay firstly in the NWP-constrained RTK. In a different way from the standard RTK, the priori wet delay $\Delta_{ZWD}$ predicted by the NWP model with a priori variance introduced into the troposphere vector in this algorithm. At the same time, a wet delay residual is estimated as an unknown parameter during the processing. Different from that of the NWP-constrained RTK, the corrected priori wet delay $\Delta_{ZWD}$ calculated by the method proposed in this study is introduced in the corrected NWP-constrained RTK processing. 

#### 4.2.2. GPS RTK Results

One day of GPS observations from five stations located in the area shown in Figure 7 were collected. The observation interval was 30 s. In this experiment, six long-range baselines between the stations were studied, and the lengths of the baselines were about 121 km, 155 km, 176 km, 193 km and 207 km, respectively. Dual-frequency GPS code and carrier phase signals were used in this experiment. GPS observation was processed every minute following the three RTK scenarios mentioned above. Least-squares AMBiguity Decorrelation Adjustment (LAMBDA) method was used as the ambiguity resolution technique [37]. The popular R-ratio is used as the validation method and a threshold value is set to 2.5 [38].

**Figure 12:**
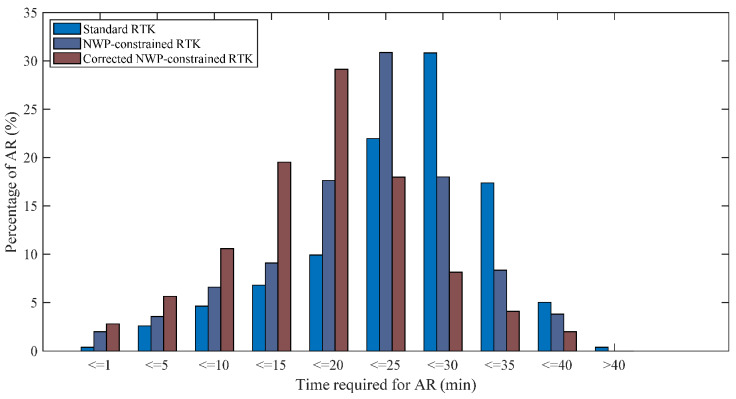
Time required for ambiguity resolution (baseline: 155 km).

**Figure 13:**
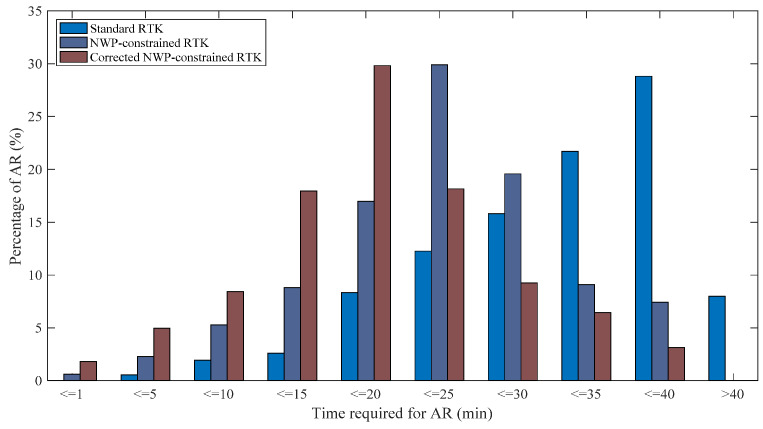
Time required for ambiguity resolution (baseline: 207 km).

Time required for initialization is an important index to measure the performance of the RTK. As an example, Figure 12 and Figure 13 illustrate the initialization time needed to fix the ambiguities for the standard RTK, the NWP-constrained RTK and the corrected NWP-constrained RTK. Table 5 presents the mean times for ambiguity resolution. For the baseline of 155 km, it is noticed that about 45%, 70% and 85% of ambiguities are fixed in 25 min with the standard RTK, the NWP-constrained RTK and the corrected NWP-constrained RTK. The mean time needed for the NWP-constrained RTK and the corrected NWP-constrained RTK is 21.5 min and 16.3 min, which is reduced by 24.8% and 43.0% compared to that of the standard RTK, respectively.

For the baseline of 207 km, the initialization required time is longer than that of the baseline of 155 km. The mean time of 36.2 min is needed for obtaining the fixed solution with standard RTK. In comparison, it takes about 22 min (mean time) for the ambiguity resolution with the NWP-constrained RTK, shortening the solution initialization time by 37.9%. More significantly, a reduction of 53.3% is revealed by the RTK constrained with corrected NWP ZTD compared with the standard RTK. The mean time needed is only 16.9 min, showing a reduction of 24.9% compared with the NWP-constrained RTK. For the other three baselines, similar results were shown in Table 5 and Table 6. As a result, the corrected NWP-constrained RTK reveals significant contribution in improving the initialization speed of RTK.

Another important index for measuring the performance of the RTK is positioning precision. Since the positioning precision of the RTK scenarios are very similar to each other after the ambiguities are fixed successfully, we only display of the results of the corrected NWP-constrained RTK. Table 6 shows the positioning precision of the baseline of 155 and 207 km in terms of the North, East and Up direction. It can be noticed that the positioning precision is better than 3 cm in the horizontal direction and better than 5 cm in the vertical direction, which satisfies the requirement of the precise positioning service.

## 5. Conclusions

To obtain a high-precision ZTD, firstly, the ZTD accuracy inverted by ECMWF and NCEP reanalysis data was compared, and then the change law of the residual of ZTD using ECMWF data (with higher accuracy compared with that inverted by NCEP data) was analyzed on the basis of temperature, humidity, latitude, and season. Afterwards, a regional NWP tropospheric delay inversion method based on a GRNN model was proposed. Finally, to evaluate the validity and accuracy of this new model, NCAR troposphere data and ECMWF data of 650 stations in Japan in 2005 were collected and analyzed. From the experiment results, we can draw some conclusions as follows:

1. The accuracy of the ZTD inverted by using the ECMWF reanalysis and by the integral method is higher than that inverted using NCEP data.
2. The ZTD residual has a high correlation with the temperature, relative humidity, latitude, and season. In general, the ZTD residual is relatively high when the temperature is high; the curves of relative humidity and the ZTD residual are similar; the accuracy of the ZTD in high latitudes is higher than that in low latitudes; the variation of the ZTD residual has an inter annual variation.
3. After using the GRNN model, the mean residual and RMSD of the ZTD of 550 test stations in Japan were found to be 9.5 mm and 12.7 mm, respectively, showing reductions of 20.8% and 19.1%, respectively.
4. For long-range baseline (155 km and 207 km), the corrected NWP-constrained RTK show a reduction of over 43% in the initialization time compared with the standard RTK, and show a reduction of over 24% in the initialization time compared with the standard NWP-constrained RTK. In addition, the positioning precisions of both long-range baselines are better than 3 cm in the horizontal direction and better than 5 cm in the vertical direction, which satisfies the requirement of the precise positioning service.

As a result, to improve the performance of the medium-/long-range baseline NRTK, we can take the data of reference stations as training data using the method proposed in this paper, and accurately estimate the tropospheric delay of the user station and the reference station that experiences tropospheric data losses due to signal blockage or interruption.

## Figures and Tables

**Table 1:** Residual and RMSD of the ZTD inverted by ECMWF and NCEP reanalysis data.

Residual/RMSD		Mean Value	Minimum Value	Maximum Value
IGS-ECMWF	Residual (mm)	13.8	6.3	26.2
	RMSD (mm)	17.1	8.1	30.9
IGS-NCEP	Residual (mm)	16.5	10.1	29.7
	RMSD (mm)	20.0	12.3	33.9

**Table 2:** Accuracy comparison of the ZTD inverted by ECMWF data at different latitudes.

Residual/RMSD		Mean Value	Minimum Value	Maximum Value
Low-latitude region	Residual (mm)	17.3	10.0	26.2
	RMSD (mm)	21.7	12.5	30.9
Mid-latitude region	Residual (mm)	13.6	7.4	21.8
	RMSD (mm)	16.9	9.6	26.4
High-latitude region	Residual (mm)	10.2	6.3	19.7
	RMSD (mm)	11.8	8.1	20.9

**Table 3:** The ZTD mean residual before and after using the GRNN model for selected stations.

Stations	Using GRNN or NOT	Mean	Minimum	Maximum
100 training stations	before GRNN	11.8	8.9	15.6
	after GRNN	8.0	5.3	10.6
550 test stations	before GRNN	12.0	8.1	18.1
	after GRNN	9.5	6.4	13.7

**Table 4:** The RMSD and SD of the residual before and after using the GRNN model for selected stations.

Stations	Using GRNN or NOT	RMSD/SD	Mean	Minimum	Maximum
100 training stations	before GRNN	RMSD (mm)	15.5	11.6	20.1
		SD (mm)	13.2	9.9	17.2
	After GRNN	RMSD (mm)	10.9	8.1	13.9
		SD (mm)	10.7	8.1	13.9
550 test stations	before GRNN	RMSD (mm)	15.7	10.6	23.1
		SD (mm)	12.9	8.6	19.3
	After GRNN	RMSD (mm)	12.7	8.5	17.7
		SD (mm)	12.4	8.3	17.5

**Table 5:** Mean times for ambiguity resolution.

Baseline	Mean Times for Ambiguity Resolution (min)		
	Standard RTK	NWP-Constrained RTK	Corrected NWP-Constrained RTK
121 Km	25.3	20.4	15.7
155 Km	28.6	21.5	16.3
176 Km	30.4	21.8	16.6
193 Km	35.1	22.2	16.7
207 Km	36.2	22.5	16.9

**Table 6:** Positioning precision of the North, East and Up directions (m).

Baseline	N	E	U
baseline: 121 km	0.015	0.013	0.031
baseline: 155 km	0.017	0.013	0.033
baseline: 176 km	0.017	0.014	0.034
baseline: 193 km	0.019	0.017	0.036
baseline: 207 km	0.021	0.018	0.039
